# Radiomics for Differentiating Prostate-Specific Membrane Antigen (PSMA)-Positive and PSMA-Negative Lymph Nodes in Prostate Cancer: An Exploratory 68Ga-PSMA PET-CT Study

**DOI:** 10.7759/cureus.114530

**Published:** 2026-08-14

**Authors:** Alejandro Hernández-Alejo, Xavier A González-Ballesteros, Haziel Maya-García, Diego Salinas-Rodríguez, Alejandro Ramírez-Rayo, Aldo M Pacheco-Carrillo, Fernando Ramírez-Trejo, Matías Salinas-Chapa, Guillermo Elizondo Riojas

**Affiliations:** 1 Radiology Department, Autonomous University of Nuevo León, "Dr. José Eleuterio González" University Hospital, Monterrey, MEX; 2 Radiology Department, San Angel Inn Hospital, Mexico City, MEX

**Keywords:** lymph nodes, prostate cancer metastasis, prostate cancer (pca), psma pet/ct, radiomic signature

## Abstract

Introduction

Accurate lymph node assessment in prostate cancer remains clinically relevant for staging and treatment planning. Although 68Ga-prostate-specific membrane antigen (PSMA) PET-CT has improved nodal evaluation, quantitative imaging biomarkers may provide additional information beyond visual assessment. This exploratory study aimed to characterize CT-derived radiomic features extracted from PSMA-positive and PSMA-negative lymph nodes using 68Ga-PSMA PET/CT positivity as the imaging reference standard.

Materials and methods

This retrospective, cross-sectional study included 41 patients with prostate cancer who underwent 68Ga-PSMA PET-CT. A total of 414 lymph nodes were segmented, including 62 PSMA-positive and 352 PSMA-negative nodes. Radiomic feature extraction was performed using 3D Slicer and pyradiomics. Twenty-nine radiomic features were evaluated and categorized into shape and texture feature families, including gray level size zone matrix, gray level run length matrix, neighboring gray tone difference matrix, and gray level dependence matrix. The descriptive comparison and diagnostic performance of all evaluated radiomic features were reported. Statistical comparisons were performed between PSMA-positive and PSMA-negative lymph nodes, and receiver operating characteristic curve analysis was used to assess discriminatory performance.

Results

Several radiomic features differed significantly between PSMA-positive and PSMA-negative lymph nodes. The highest discriminatory performance was observed for long run high gray level emphasis, with an area under the curve of 0.763, sensitivity of 77.42%, and specificity of 67.90%. Other high-performing features included high gray level emphasis, with an area under the curve of 0.751; large dependence high gray level emphasis, with an area under the curve of 0.746; high gray level run emphasis, with an area under the curve of 0.744; short run high gray level emphasis, with an area under the curve of 0.731; and low gray level emphasis, with an area under the curve of 0.726.

Conclusion

Radiomic features extracted from 68Ga-PSMA PET-CT differed between PSMA-positive and PSMA-negative lymph nodes and demonstrated moderate discriminatory performance. The highest-performing features were predominantly texture-based metrics, suggesting that radiomic analysis may provide complementary quantitative information for lymph node characterization in prostate cancer. However, given the exploratory design, class imbalance, manual segmentation, lack of individual-node histopathological confirmation, and absence of external validation, these results should be interpreted as hypothesis-generating. Future prospective studies with standardized segmentation, histopathological correlation, and multivariable model validation are needed before clinical implementation.

## Introduction

Prostate cancer (PCa) is one of the most common malignancies among men worldwide and remains a major cause of cancer-related mortality. Globally, it is the second most frequently diagnosed cancer and the fifth leading cause of cancer death among men [1,2]. Precise staging of prostate cancer plays a critical role in determining appropriate initial therapy. The identification of pelvic lymph node involvement during radical prostatectomy has been linked to a higher likelihood of biochemical relapse and is an important prognostic factor that influences staging, treatment planning, and prognosis [3].

To aid in accurate staging, current guidelines from the European Association of Urology recommend at least one cross-sectional imaging study of the abdomen and pelvis, along with a bone scan, for staging locoregional and metastatic disease in prostate cancer patients [4,5]. Abdominal computed tomography and magnetic resonance imaging have sensitivities of 42% and 39% and specificities of 82% and 82%, respectively, for the detection of nodal metastases [4]. This limitation is largely attributable to its reliance on nodal size and morphology: microscopic metastases may occur in normal-sized lymph nodes, whereas benign or reactive nodes may be enlarged, resulting in substantial overlap between malignant and non-malignant appearances [4,6].

One promising molecular target is prostate-specific membrane antigen (PSMA), a transmembrane protein with an extracellular portion consisting of 707 amino acids. The PSMA gene is located on the short arm of chromosome 11 and is expressed in the apical region of prostate cells. Dysplastic changes in the prostate, as well as cancer progression, lead to the overexpression of PSMA on the luminal surface of prostate ducts, resulting in a 100 to 1000-fold higher PSMA expression on the cell membrane compared to normal cells [7,8].

Importantly, multiple studies have demonstrated that PSMA PET/CT provides superior performance for lymph node and metastatic staging compared with conventional imaging modalities, including MRI, contrast-enhanced CT, and choline PET/CT [9]. In particular, 68Ga-PSMA PET/CT has shown greater sensitivity for detecting metastatic lymph nodes and nodal recurrence than morphology-based CT or MRI assessment, while maintaining high specificity [10,11].

Moreover, 68Ga-PSMA PET-CT has a detection capability of over 90% for lymph nodes measuring 4.5 mm or larger, allowing it to detect metastatic lymph nodes earlier than computed tomography or magnetic resonance imaging, which typically require a size of at least 8 mm [4,12].

However, despite the high diagnostic value of 68Ga-PSMA PET-CT in cases of biochemical recurrence, approximately 40% of patients with a PSA level less than 0.5 ng/mL may yield false-negative results. Certain cutoff points are established, such as a PSA level of 0.83 ng/mL and a doubling of serum PSA levels within 6.5 months, which indicate a high probability of negative or positive results [11,13]. In this context, the high specificity of 68Ga-PSMA PET-CT (up to 99%) is particularly valuable, especially given the high morbidity associated with radical lymphadenectomy [4,11].

To address these challenges, radiomics has emerged as a comprehensive and automated method for quantifying the radiographic phenotype, utilizing data characterization algorithms [14-16] that allow for the quantification of a multitude of phenotypic features (radiomic signature), such as shape and texture, which potentially reflect biological properties including intra- and inter-tumoral heterogeneities [17].

Previous studies indicate that radiomics-based analysis performs at least as well as standard PET metrics and can predict lymph node involvement and high-risk pathological tumor features in patients with primary PCa, while also offering the advantage of no additional cost, since radiomic features are derived from the same analysis pipeline as conventional metrics [18].

Nonetheless, radiomics is currently limited by several challenges that restrict its clinical translation. Among the most critical are the scarcity of prospective studies and the lack of robust external validation for existing models. These shortcomings prevent radiomics from evolving beyond a research-focused discipline into a clinically valuable tool capable of guiding personalized treatment based on individual tumor characteristics. Addressing these issues is essential to fully realize the clinical value of radiomics [19].

The distinctive contribution of this study is its direct lesion-level evaluation of CT-derived radiomic features extracted from the lymph nodes themselves. Unlike studies that derive radiomic information from the primary prostate tumor or construct a multivariable predictive model, this study characterized the discriminatory performance of individual lymph-node features using PSMA PET/CT positivity as the imaging reference standard. Furthermore, the CT component was selected for radiomic analysis because it is routinely acquired as part of PSMA PET/CT, provides quantitative anatomical and attenuation information, and can be analyzed without requiring an additional imaging examination or additional radiation exposure.

Accordingly, this exploratory study aimed to characterize CT-derived radiomic features of PSMA-positive and PSMA-negative lymph nodes in patients with prostate cancer and to assess the discriminatory performance of the evaluated features using 68Ga-PSMA PET/CT positivity as the imaging reference standard.

## Materials and methods

Methodological design

This retrospective, cross-sectional, descriptive study included 41 randomly selected patients with pathologically confirmed prostate cancer who underwent 68Ga-PSMA PET/CT at the University Diagnostic Imaging Center of "Dr. José E. González" University Hospital between February 2021 and February 2022. The target sample of 41 patients was established a priori. Eligible examinations were selected using a random-number sequence generated through Random.org. Imaging studies were anonymized using consecutive alphanumeric codes before analysis.

Group stratification and lymph node selection process

Patients were divided into two groups (group A and group B). Group A included patients with at least one lymph node positive for prostate cancer by 68Ga-PSMA, while group B included patients with negative findings for malignant lymph nodes by 68Ga-PSMA.

In group A, one lymph node per region (right and left in the case of pelvic, axillary, and cervical chains) and two adjacent lymph nodes at the retroperitoneal and mediastinal levels were selected. We chose nodes with the highest radioisotope uptake (highest SUVmax) and their respective contralateral nodes. In cases where no nodes showed positive radioisotope uptake, we segmented the largest node (short axis).

In group B, one lymph node per region (right and left in the case of pelvic, axillary, and cervical chains) and two adjacent lymph nodes at the retroperitoneal and mediastinal levels were selected. We chose the largest nodes (short axis).

For the lesion-level comparative analysis, the analytic cohort included all PSMA-positive lymph nodes from group A and all selected lymph nodes from group B; PSMA-negative lymph nodes from group A were omitted. This yielded 414 analyzed lymph nodes (62 PSMA-positive (15.0%) and 352 PSMA-negative (85.0%)). This sampling rule was used to standardize lesion selection across patients and anatomical regions and to limit disproportionate representation of patients with numerous visible lymph nodes. However, prioritizing the most avid or largest nodes may preferentially select more conspicuous lesions and is therefore acknowledged as a potential source of selection bias.

Lymph nodes from both groups were manually segmented on CT images using 3D Slicer [20], and radiomic features were extracted using PyRadiomics [21]. A database was generated in SPSS (IBM Inc., Armonk, NY, USA) for statistical analysis to evaluate which radiomic features best differentiated PSMA-positive from PSMA-negative regional and non-regional lymph nodes, using 68Ga-PSMA PET/CT positivity as the imaging reference standard.

Analyzed variables

Twenty-nine CT-derived radiomic features were analyzed according to standardized PyRadiomics and Image Biomarker Standardization Initiative definitions [21,22]. These comprised four shape features and 25 texture features derived from the gray level size zone matrix, gray level run length matrix, neighboring gray tone difference matrix, and gray level dependence matrix families.

Inclusion criteria

To be eligible for participation in this study, individuals must meet specific criteria. Firstly, participants should be at least 18 years of age. Secondly, patients must have a confirmed diagnosis of prostate cancer, which has been established through pathological examination. Lastly, eligible patients were required to have retrievable 68Ga-PSMA PET/CT examinations from the radiology department at "Dr. José E. González" University Hospital.

Exclusion criteria

Patients were excluded when the imaging examination could not be retrieved or when another active malignancy was documented. Previous pelvic radiotherapy, systemic therapy, and prior lymphadenectomy were not prespecified exclusion criteria; therefore, patients with these treatment histories may have been included. Because these treatment variables were not systematically captured in the analytic database, their potential effects on lymph-node morphology and radiomic features could not be evaluated.

Elimination criteria

Segmented lymph nodes were eliminated from the final radiomic analysis when the segmented lesion was not considered suitable for reliable radiomic feature extraction. This included poor image quality, severe motion or attenuation-correction artifacts, inadequate lesion delineation, incomplete inclusion of the target lymph node, substantial contamination from adjacent structures, a small size or partial-volume effect that prevented reproducible segmentation, inconsistent identification across PET-CT images, or technical limitations that prevented adequate radiomic processing.

Radiomic feature extraction

Lymph nodes were segmented, and the database was generated using 3D Slicer software [20] to extract radiomic features from PSMA-positive and PSMA-negative lymph nodes identified in 68Ga-PSMA PET/CT examinations of patients with prostate cancer performed at the University Diagnostic Imaging Center of "Dr. José E. González" University Hospital. All examinations were acquired using the same contrast-enhanced diagnostic CT protocol on a Discovery 690 Elite PET/CT system (GE Healthcare) equipped with a 16-detector-row CT component. CT acquisition extended from the vertex to the mid-thigh and was performed in the venous phase, 45 seconds after the administration of a nonionic iodinated contrast agent (iopromide, 1 mL/kg). Images were acquired helically at 120 kVp using automatic tube-current modulation with a noise index of 28.5, a gantry rotation time of 0.8 seconds, a pitch of 1.375:1, and no gantry tilt. The CT images used for radiomic analysis were reconstructed with a 2.5-mm section thickness, a standard soft-tissue reconstruction kernel (STND), a 512 × 512 matrix, and a 70-cm display field of view, corresponding to an approximate in-plane pixel size of 1.37 × 1.37 mm. The exact software versions, original PyRadiomics preprocessing configuration, and reader-experience information were not preserved in the available records and could not be reconstructed retrospectively.

Sample size calculation

A single-proportion calculation was performed at the patient level using n = Z²p(1-p)/d². A two-sided 95% confidence level was specified (Z = 1.96), with an expected 68Ga-PSMA PET positivity proportion of 0.40 based on the meta-analysis by Perera et al. [23] and an absolute precision of 0.15, corresponding to a total confidence-interval width of 0.30. The calculated sample size was 40.98 and was rounded up to 41 patients.

Statistical analysis

Descriptive statistics were used to summarize continuous variables, which are reported as mean ± standard deviation (SD). Comparisons between PSMA-positive and PSMA-negative lymph nodes were performed using independent-samples Student's t-tests or Welch's t-tests, as appropriate. Receiver operating characteristic (ROC) curve analysis was performed to evaluate the discriminatory performance of radiomic features. The area under the ROC curve (AUC) was tested against the null value of 0.5 using a z statistic. No correction for multiple comparisons was applied; therefore, findings should be interpreted as exploratory. Analyses were performed using SPSS version 25.0 (IBM Inc., Armonk, NY, USA), with statistical significance set at p < 0.05.

## Results

Analyzed variables

Forty-one patients diagnosed with prostate cancer who underwent 68Ga-PSMA PET-CT were included. A total of 414 lymph nodes were analyzed, including 62 PSMA-positive nodes (15.0%) and 352 PSMA-negative nodes (85.0%). Twenty-nine radiomic features were evaluated and categorized into shape, gray level size zone matrix, gray level run length matrix, neighboring gray tone difference matrix, and gray level dependence matrix.

Radiomic features of PSMA-positive and PSMA-negative lymph nodes

Among the analyzed radiomic features, PSMA-positive lymph nodes showed higher values for major axis length than PSMA-negative nodes (20.95 ± 13.45 vs. 15.50 ± 7.25; p = 0.003). Size zone non-uniformity was also higher in PSMA-positive nodes (11.13 ± 19.81 vs. 4.86 ± 3.36; p = 0.016), as were long run high gray level emphasis (67.57 ± 62.06 vs. 35.24 ± 17.93; p < 0.001), high gray level run emphasis (29.98 ± 23.06 vs. 19.48 ± 8.08; p < 0.001), short run high gray level emphasis (24.74 ± 19.57 vs. 16.69 ± 6.80; p = 0.002), high gray level emphasis (30.05 ± 15.76 vs. 19.88 ± 8.64; p < 0.001), and large dependence high gray level emphasis (2038.95 ± 1928.51 vs. 837.12 ± 719.11; p < 0.001). Conversely, low gray level emphasis was lower in PSMA-positive nodes than in PSMA-negative nodes (0.09 ± 0.06 vs. 0.14 ± 0.07; p < 0.001). The descriptive results of all 29 evaluated features are presented in Table 1.

**Table 1:** Radiomic features analyzed in PSMA-positive and PSMA-negative lymph nodes, expressed as mean ± standard deviation Comparisons used independent-samples Student’s t-tests or Welch’s t-tests, as identified in the Test statistic column. Statistical significance was set at p < 0.05. PSMA - prostate-specific membrane antigen

Feature	PSMA-positive nodes (n=62; 15.0%)	PSMA-negative nodes (n=352; 85.0%)	Test statistic	p-value
Shape				
Major axis length	20.95 ± 13.45	15.50 ± 7.25	Welch t(67.4) = 3.109	0.003
Sphericity	0.75 ± 0.07	0.78 ± 0.06	Student t(412) = -3.622	<0.001
Surface area	854.19 ± 963.37	569.12 ± 503.97	Welch t(67.0) = 2.276	0.026
Minor axis length	11.49 ± 4.53	10.03 ± 3.95	Student t(412) = 2.615	0.009
Gray level size zone matrix				
Gray level non-uniformity normalized	0.18 ± 0.04	0.19 ± 0.04	Student t(412) = -1.994	0.047
Size zone non-uniformity	11.13 ± 19.81	4.86 ± 3.36	Welch t(61.6) = 2.487	0.016
Small area high gray level emphasis	10.48 ± 18.30	6.35 ± 3.50	Student t(412) = 3.877	<0.001
Small area emphasis	0.46 ± 0.09	0.41 ± 0.11	Student t(412) = 3.201	0.001
Low gray level zone emphasis	0.19 ± 0.08	0.24 ± 0.11	Student t(412) = -3.532	<0.001
Zone entropy	4.16 ± 0.70	3.78 ± 0.63	Student t(412) = 4.315	<0.001
Gray level run length matrix				
Short run low gray level emphasis	0.09 ± 0.05	0.13 ± 0.06	Student t(412) = -4.548	<0.001
Low gray level run emphasis	0.10 ± 0.06	0.14 ± 0.07	Student t(412) = -4.567	<0.001
Gray level non-uniformity normalized	0.18 ± 0.05	0.19 ± 0.04	Student t(412) = -2.279	0.023
Gray level non-uniformity	57.10 ± 72.05	40.83 ± 50.12	Student t(412) = 2.191	0.029
Short run high gray level emphasis	24.74 ± 19.57	16.69 ± 6.80	Welch t(63.6) = 3.207	0.002
Short run emphasis	0.86 ± 0.05	0.87 ± 0.04	Student t(412) = -2.602	0.01
Long run high gray level emphasis	67.57 ± 62.06	35.24 ± 17.93	Welch t(62.8) = 4.072	<0.001
Run percentage	0.81 ± 0.08	0.84 ± 0.06	Welch t(71.7) = -2.685	0.009
High gray level run emphasis	29.98 ± 23.06	19.48 ± 8.08	Welch t(63.7) = 3.546	<0.001
Run length non-uniformity normalized	0.70 ± 0.08	0.73 ± 0.07	Student t(412) = -2.695	0.007
Neighboring gray tone difference matrix				
Coarseness	0.04 ± 0.05	0.06 ± 0.06	Welch t(93.8) = -3.074	0.003
Busyness	1.28 ± 1.10	1.70 ± 2.03	Welch t(147.4) = -2.406	0.017
Gray level dependence matrix				
High gray level emphasis	30.05 ± 15.76	19.88 ± 8.64	Welch t(67.6) = 4.951	<0.001
Dependence entropy	5.36 ± 0.59	5.05 ± 0.72	Welch t(96.7) = 3.690	<0.001
Large dependence emphasis	54.06 ± 48.22	37.35 ± 22.48	Welch t(65.7) = 2.677	0.009
Dependence variance	14.17 ± 14.98	8.00 ± 6.08	Welch t(64.6) = 3.198	0.002
Large dependence high gray level emphasis	2038.95 ± 1928.51	837.12 ± 719.11	Welch t(64.0) = 4.848	<0.001
Small dependence low gray level emphasis	0.02 ± 0.01	0.03 ± 0.02	Welch t(113.7) = -3.091	0.003
Low gray level emphasis	0.09 ± 0.06	0.14 ± 0.07	Student t(412) = -4.598	<0.001

Performance indicators of shape and texture radiomic features

**Figure 1:**
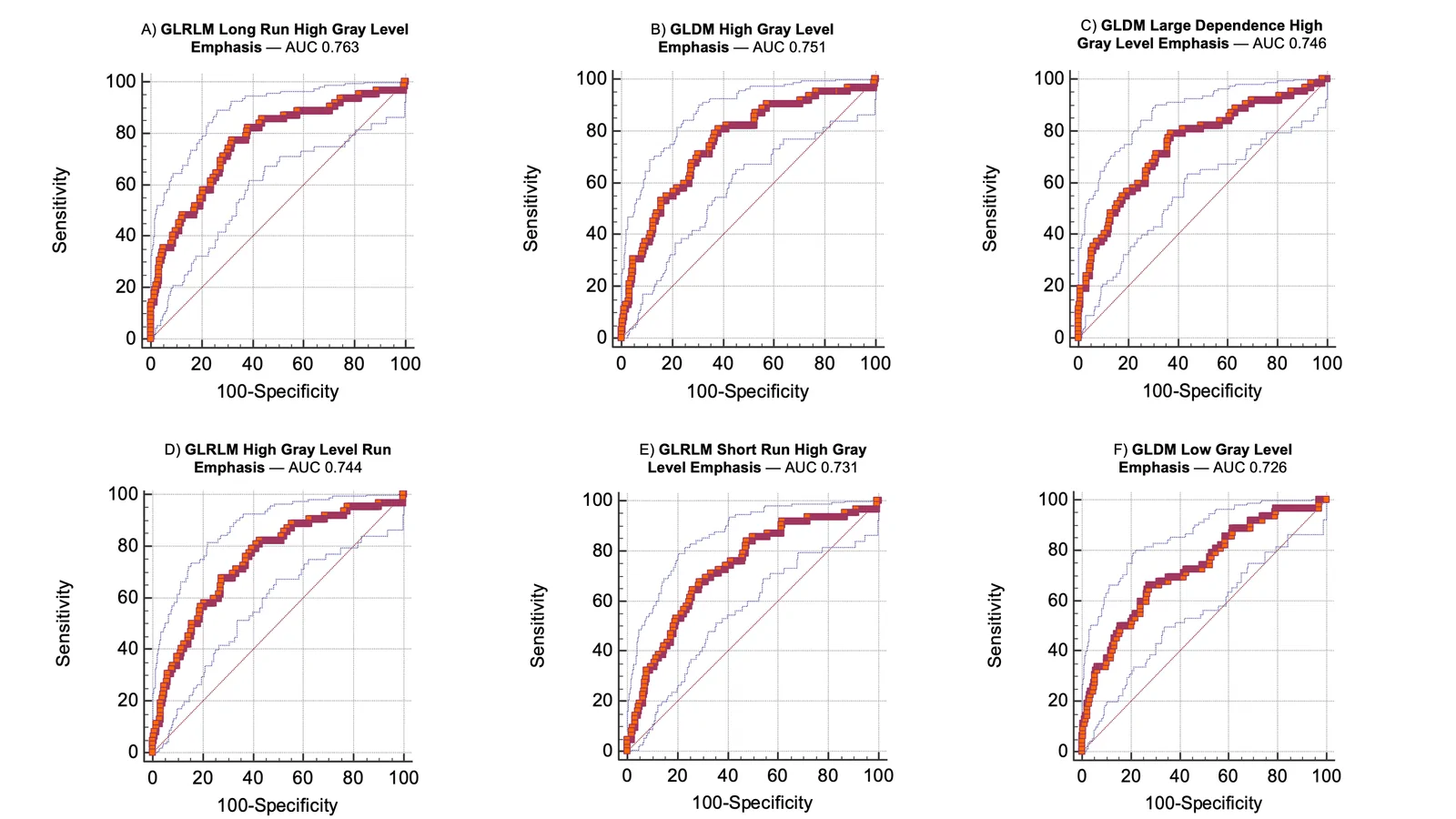
Receiver operating characteristic (ROC) curves of the six radiomic features with the highest discriminatory performance for differentiating PSMA-positive and PSMA-negative lymph nodes A) GLRLM long run high gray level emphasis; B) GLDM high gray level emphasis; C) GLDM large dependence high gray level emphasis; D) GLRLM high gray level run emphasis; E) GLRLM short run high gray level emphasis; F) GLDM low gray level emphasis The diagonal line represents the line of no discrimination. ROC - receiver operating characteristic; PSMA - prostate-specific membrane antigen; GLRLM - gray level run length matrix; GLDM - gray level dependence matrix

**Figure 2:**
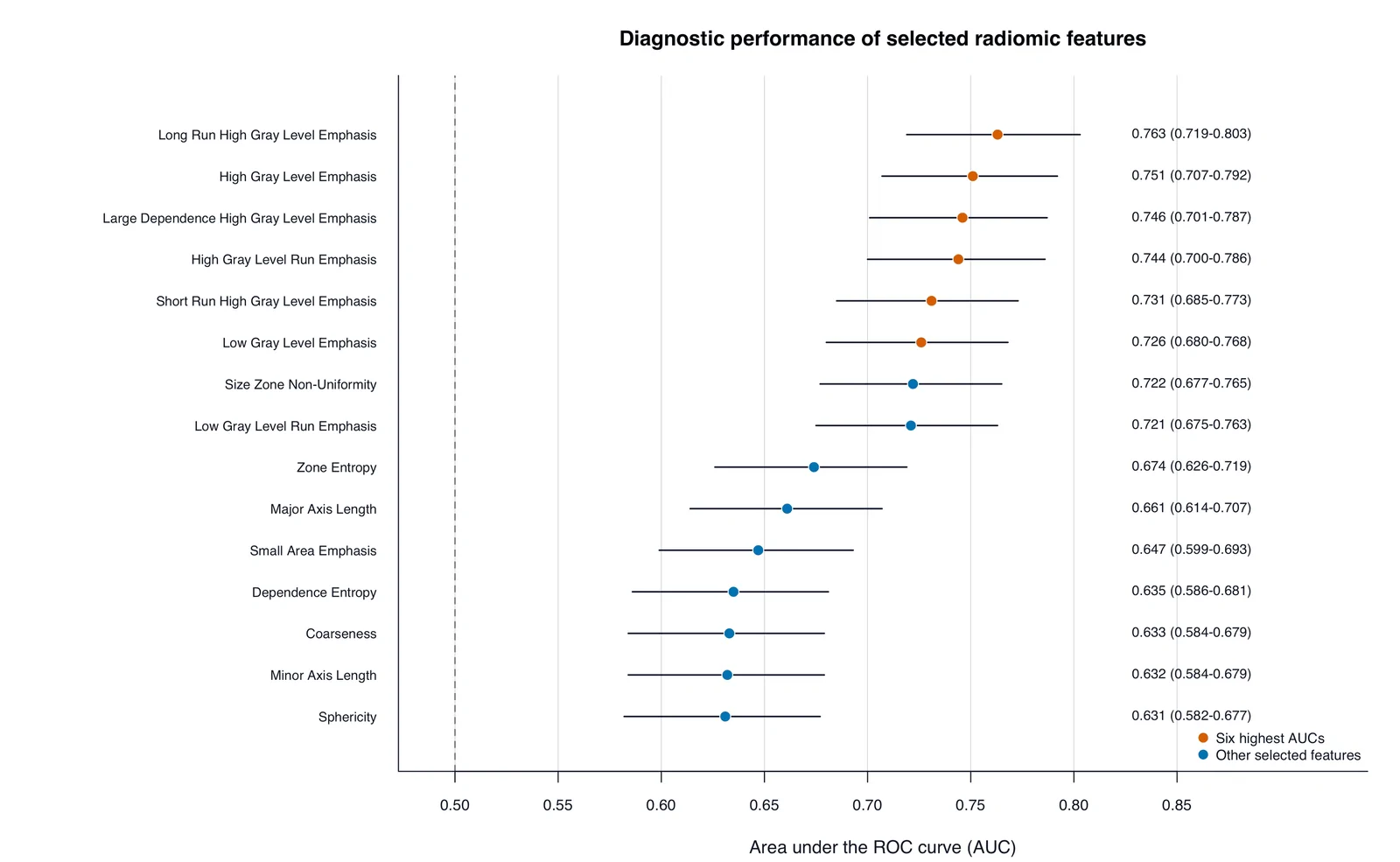
Forest plot of the area under the receiver operating characteristic curve (AUC) and corresponding 95% confidence intervals (CIs) for 15 selected radiomic features used to differentiate PSMA-positive from PSMA-negative lymph nodes Orange symbols identify the six features with the highest AUC values shown in Figure 1, and blue symbols represent the remaining features. The dashed vertical line at AUC = 0.50 indicates no discrimination. Analyses included 414 lymph nodes: 62 PSMA-positive (15.0%) and 352 PSMA-negative (85.0%). GLDM - gray level dependence matrix; GLRLM - gray level run length matrix; GLSZM - gray level size zone matrix; NGTDM - neighboring gray tone difference matrix; PSMA - prostate-specific membrane antigen

ROC analysis showed that long run high gray level emphasis had the highest discriminatory performance, with an AUC of 0.763 (95% CI: 0.719-0.803), sensitivity of 77.42%, and specificity of 67.90%. High gray level emphasis showed an AUC of 0.751 (95% CI: 0.707-0.792), sensitivity of 80.65%, and specificity of 61.65%. Large dependence high gray level emphasis demonstrated an AUC of 0.746 (95% CI: 0.701-0.787), sensitivity of 79.03%, and specificity of 62.78%. High gray level run emphasis showed an AUC of 0.744 (95% CI: 0.700-0.786), sensitivity of 67.74%, and specificity of 72.44%. Short run high gray level emphasis showed an AUC of 0.731 (95% CI: 0.685-0.773), sensitivity of 67.74%, and specificity of 71.02%. Low gray level emphasis showed an AUC of 0.726 (95% CI: 0.680-0.768), sensitivity of 66.13%, and specificity of 72.44%. These six features showed the highest AUC values among the 29 evaluated variables and were selected for graphical representation in Figure 1. The diagnostic performance of all evaluated radiomic features is presented in Table 2. Figure 2 summarizes the AUC estimates and 95% confidence intervals for the 15 best-performing radiomic features, demonstrating that texture-related metrics accounted for the highest estimates, whereas shape features and the remaining texture features showed lower but still above-chance discrimination.

**Table 2:** Diagnostic performance of radiomic features for differentiating PSMA-positive and PSMA-negative lymph nodes ROC analyses included 414 lymph nodes: 62 PSMA-positive (15.0%) and 352 PSMA-negative (85.0%). Values are the optimal criterion, area under the ROC curve (AUC) with 95% confidence interval (CI), z statistic testing the AUC against 0.5, Youden index J [24], sensitivity, specificity, and p-value. Statistical significance was set at p < 0.05. ROC - receiver operating characteristic; PSMA - prostate-specific membrane antigen

Feature	Associated criterion	Area under the ROC curve (95% CI)	z statistic	Youden index J [24]	Sensitivity (%)	Specificity (%)	p-value
Shape							
Major axis length	>13.4513	0.661 (0.614-0.707)	4.608	0.2932	79.03	50.28	<0.0001
Sphericity	≤0.7516	0.631 (0.582-0.677)	3.316	0.2852	58.06	70.45	0.0009
Surface area	>436.1228	0.651 (0.603-0.697)	4.27	0.2883	72.58	56.25	<0.0001
Minor axis length	>9.5244	0.632 (0.584-0.679)	3.656	0.2949	70.97	58.52	0.0003
Gray level size zone matrix							
Gray level non-uniformity normalized	≤0.1799	0.557 (0.508-0.605)	1.48	0.1461	62.9	51.7	0.1388
Size zone non-uniformity	>5.3077	0.722 (0.677-0.765)	5.951	0.383	70.97	67.33	<0.0001
Small area high gray level emphasis	>6.8903	0.633 (0.584-0.679)	3.351	0.2436	61.29	63.07	0.0008
Small area emphasis	>0.427	0.647 (0.599-0.693)	4.082	0.3044	74.19	56.25	<0.0001
Low gray level zone emphasis	≤0.154	0.647 (0.599-0.693)	3.792	0.2196	43.55	78.41	0.0001
Zone entropy	>4.162	0.674 (0.626-0.719)	4.833	0.3155	59.68	71.87	<0.0001
Gray level run length matrix							
Short run low gray level emphasis	≤0.083	0.719 (0.673-0.762)	5.989	0.3515	61.29	73.86	<0.0001
Low gray level run emphasis	≤0.0934	0.721 (0.675-0.763)	6.074	0.3734	62.9	74.43	<0.0001
Gray level non-uniformity normalized	≤0.1788	0.619 (0.570-0.666)	3.135	0.3063	70.97	59.66	0.0017
Gray level non-uniformity	>21.5449	0.632 (0.584-0.679)	3.696	0.2609	75.81	50.28	0.0002
Short run high gray level emphasis	>19.239	0.731 (0.685-0.773)	6.572	0.3876	67.74	71.02	<0.0001
Short run emphasis	≤0.8719	0.597 (0.548-0.644)	2.54	0.2275	64.52	58.24	0.0111
Long run high gray level emphasis	>36.633	0.763 (0.719-0.803)	7.45	0.4532	77.42	67.9	<0.0001
Run percentage	≤0.8366	0.610 (0.561-0.657)	2.87	0.2332	64.52	58.81	0.0041
High gray level run emphasis	>23.0185	0.744 (0.700-0.786)	6.973	0.4019	67.74	72.44	<0.0001
Run length non-uniformity normalized	≤0.7199	0.602 (0.553-0.650)	2.682	0.2389	64.52	59.38	0.0073
Neighboring gray tone difference matrix							
Coarseness	≤0.0662	0.633 (0.584-0.679)	3.746	0.2593	88.71	37.22	0.0002
Busyness	>1.7636	0.503 (0.454-0.553)	0.093	0.1162	14.52	73.86	0.9262
Gray level dependence matrix							
High gray level emphasis	>20.3535	0.751 (0.707-0.792)	7.237	0.4229	80.65	61.65	<0.0001
Dependence entropy	>4.8938	0.635 (0.586-0.681)	3.82	0.2582	85.48	40.34	0.0001
Large dependence emphasis	>33.1857	0.627 (0.578-0.673)	3.311	0.2475	69.35	55.4	0.0009
Dependence variance	>8.2943	0.662 (0.614-0.708)	4.276	0.2578	61.29	64.49	<0.0001
Large dependence high gray level emphasis	>782.8364	0.746 (0.701-0.787)	6.763	0.4182	79.03	62.78	<0.0001
Small dependence low gray level emphasis	≤0.0159	0.601 (0.552-0.648)	2.589	0.1694	46.77	70.17	0.0096
Low gray level emphasis	≤0.0918	0.726 (0.680-0.768)	6.169	0.3857	66.13	72.44	<0.0001

## Discussion

In this study, radiomic features extracted from lymph node segmentation on 68Ga-PSMA PET-CT scans differentiated PSMA-positive from PSMA-negative lymph nodes in patients with prostate cancer. The most relevant findings involved texture-related metrics, particularly long run high gray level emphasis, high gray level emphasis, large dependence high gray level emphasis, high gray level run emphasis, short run high gray level emphasis, and low gray level emphasis.

Peeken et al. [25] evaluated 80 patients and 149 lymph nodes in a PSMA radioguided surgery cohort, with histopathological confirmation available for all resected lymph nodes. Their combined CT-based radiomic model achieved the best diagnostic performance, outperforming conventional CT parameters such as short-axis diameter, lymph node volume, and expert visual assessment. Although their model incorporated texture, shape, intensity, and local binary pattern features, local binary pattern features showed the greatest contribution to model performance. This aligns with our findings by supporting the incremental value of radiomic analysis over conventional morphologic assessment for nodal characterization. In our study, shape-related features such as major axis length differed significantly between PSMA-positive and PSMA-negative lymph nodes; however, the highest discriminatory performance was observed for texture-related metrics, particularly long run high gray level emphasis, high gray level emphasis, and large dependence high gray level emphasis.

Seidler et al. [6] applied dual-energy CT texture analysis combined with machine learning algorithms to differentiate metastatic, inflammatory, and normal lymph nodes in head and neck cancers, analyzing 412 cervical nodes across five patient groups. Using histopathological confirmation and size-based criteria, they demonstrated that radiomic texture features could distinguish lymph node pathologies with high accuracy, achieving AUC values of up to 0.97 in their predictive model. Although their clinical context differs from ours, the use of texture features and ROC analysis is comparable. In contrast to their approach, we analyzed each radiomic feature individually rather than constructing a predictive model, with our best-performing feature, long run high gray level emphasis, achieving an AUC of 0.763, sensitivity of 77.42%, and specificity of 67.90%. Other texture-related features also showed moderate discriminatory performance, including high gray level emphasis, large dependence high gray level emphasis, high gray level run emphasis, short run high gray level emphasis, and low gray level emphasis.

Giesel et al. [26] evaluated lymph nodes using PET-CT-based nodal staging and semiautomated CT density analysis. In prostate cancer patients, nodal assessment was performed in the context of PSMA PET-CT. Their findings suggest that CT-density parameters may provide additional information for nodal characterization; however, their work was not limited exclusively to prostate cancer and did not focus on a broad lesion-based radiomic texture analysis. In contrast, our study focused exclusively on prostate cancer and used 68Ga-PSMA PET-CT positivity as the imaging reference standard. In addition, our analysis incorporated lesion-based radiomic features, including second-order texture metrics and shape descriptors extracted through manual segmentation using open-source software. While short-axis diameter was considered during segmentation, the highest discriminatory performance in our study was observed for texture-related metrics rather than conventional morphologic descriptors.

While previous studies, such as that of Cysouw et al. [18], obtained radiomic features from the primary tumor to indirectly predict the probability of lymph node involvement with AUC values as high as 0.86, our approach is distinct in that radiomic features were extracted directly from lymph nodes classified as PSMA-positive or PSMA-negative, allowing for a more targeted and lesion-specific analysis. This methodological difference may offer a more precise characterization of nodal disease, particularly when visual interpretation is equivocal. In our study, long run high gray level emphasis and high gray level emphasis demonstrated AUCs of 0.763 and 0.751, respectively, which are within the range of moderate discriminatory performance reported in exploratory radiomics literature.

The study by Zamboglou et al. [27] highlights an important translational perspective in the use of PSMA PET-derived radiomic features as non-invasive imaging biomarkers capable of reflecting histopathological properties such as tumor aggressiveness and nodal involvement. Their findings, validated across both prospective and retrospective cohorts, emphasize the potential robustness of texture analysis and its reproducibility across different data sources. Although our study lacked histopathological correlation at the individual-node level, our results complement this translational perspective by showing that selected lesion-based radiomic metrics can differentiate PSMA-positive from PSMA-negative lymph nodes in an exploratory setting. These observations support future multicenter studies combining lesion-based radiomics with histological validation and standardized acquisition protocols.

Although our approach relied on individual variable analysis rather than predictive modeling, several features showed moderate discriminatory performance, with the highest AUCs observed for long run high gray level emphasis, high gray level emphasis, large dependence high gray level emphasis, high gray level run emphasis, short run high gray level emphasis, and low gray level emphasis. These findings suggest that selected texture-based metrics may provide complementary quantitative information for nodal assessment, particularly as hypothesis-generating biomarkers for future multivariable models.

However, it is essential to acknowledge several limitations inherent in our retrospective study. Firstly, the manual segmentation of lymph nodes, a necessary step in our radiomic analysis, introduces potential subjectivity and variability. Additionally, the notable imbalance between positive (n=62) and negative (n=352) lymph nodes may affect the generalizability of the discriminatory thresholds identified and should be accounted for in future validation studies. Additionally, the retrospective nature of our study design introduces inherent biases, and the lack of a standardized segmentation protocol may affect the consistency of our results. Furthermore, the analysis of multiple radiomic features without correction for multiple comparisons increases the risk of type I error; findings should therefore be interpreted as hypothesis-generating rather than confirmatory. While our study provides valuable insights into the potential of radiomic analysis for lymph node characterization in prostate cancer, these limitations underscore the need for prospective studies with standardized segmentation protocols and larger sample sizes to enhance the reliability and generalizability of our results

## Conclusions

This exploratory study identified CT-derived radiomic features from 68Ga-PSMA PET-CT that differed between PSMA-positive and PSMA-negative lymph nodes in patients with prostate cancer. Among the 29 evaluated features, long run high gray level emphasis, high gray level emphasis, large dependence high gray level emphasis, high gray level run emphasis, short run high gray level emphasis, and low gray level emphasis showed the highest discriminatory performance, with AUC values ranging from 0.726 to 0.763. These findings suggest that texture-based radiomic features may provide complementary quantitative information for lymph node characterization beyond conventional visual assessment. However, given the retrospective design, manual segmentation, class imbalance, lack of histopathological confirmation at the individual-node level, and absence of external validation, these results should be interpreted as hypothesis-generating. Future prospective studies with standardized segmentation protocols, histopathological correlation, and multivariable model validation are needed before clinical implementation.
